# Effect of kegel pelvic floor muscle exercise on improving urinary disorder in rectum cancer patients after rectal surgery: a randomized clinical trial

**DOI:** 10.1007/s00384-024-04738-0

**Published:** 2024-10-21

**Authors:** Mehran Ebrahimi Shah-abadi, Haleh Pak, Alireza Kazemeini, Dorsa Najari, Seyed Mohsen Ahmadi Tafti, Mohammad Reza Keramati, Amir Keshvari, Mohammad Sadegh Fazeli, Behnam Behboudi

**Affiliations:** 1 Division of Colorectal Surgery, Department of Surgery, Imam-Khomeini Hospital Complex, Tehran University of Medical Sciences, Tehran, Iran; 2 Colorectal Research Center, Tehran University of Medical Sciences, Tehran, Iran; 3 Department of Surgery, Afzalipour Hospital, Kerman University of Medical Sciences, Kerman, Iran; 4 Department of Surgery, Alborz University of Medical Sciences, Karaj, Iran; 5 School of Medicine, Shahid Beheshti University of Medical Sciences, Tehran, Iran

**Keywords:** Rectal cancer, Quality of life, Pelvic floor muscle training (PFMT), Urinary dysfunction, Kegel exercises, Post operative care

## Abstract

**Introduction:**

Postoperative urinary dysfunction poses a significant challenge for rectal cancer patients. While pelvic floor muscle training (PFMT) has shown promise in other contexts, its efficacy following rectal cancer surgery remains uncertain.

**Results:**

A clinical trial involving 79 rectal cancer patients found that initiating Kegel exercises post-surgery led to significant improvements in urinary symptoms compared to standard care. Adherence to exercises correlated with symptom reduction, with no reported adverse events. We have defined the main outcome of our study as the improvement in urinary function scores post-surgery. Effectiveness is considered as any statistically significant improvement in these scores.

**Conclusion:**

Early initiation of Kegel exercises shows promise in alleviating postoperative urinary dysfunction in rectal cancer patients. Further research is needed to optimize postoperative care protocols and enhance patient outcomes.

## Introduction

With the increasing survival rates of rectal cancer patients post-surgery, the significance of the postoperative period has become more pronounced. Colorectal cancer (CRC) now ranks as the third leading cause of cancer-related mortality worldwide, with escalating rates particularly evident in developing countries [1]. The evolution of rectal surgery in recent decades, transitioning from abdominoperineal resection (APR) with permanent colostomy to advanced techniques like total mesorectal excision (TME) and sphincter-saving procedures, underscores the progress in surgical approaches [2].

As a considerable number of patients now achieve long-term survival, the prevention of postoperative complications assumes paramount importance. It is well-established that surgical interventions can disrupt normal organ function and anatomy, particularly affecting pelvic organs and leading to bowel, urogenital, and sexual dysfunctions [3]. While urinary complications following rectal resections are relatively less frequent and severe compared to bowel issues, their impact on quality of life can be substantial, potentially diminishing it by up to 30% [3]. The etiology of urogenital dysfunction post-rectal cancer excision is predominantly attributed to surgical factors, notably autonomic nerve injuries involving the inferior hypogastric plexus or pelvic splanchnic nerves due to disruptions in the avascular plane between visceral and parietal pelvic fascia [4, 5].

Extensive research has explored the benefits of pelvic floor muscle training, such as Kegel exercises, post-prostatectomy to mitigate urinary incontinence and erectile dysfunction. However, despite sharing a common etiology, the effectiveness of Kegel exercises following rectal cancer surgery remains uncertain [6, 7]. Therefore, this clinical trial aims to investigate the impact of these exercises on urinary dysfunction subsequent to colorectal surgery, marking the first comprehensive evaluation of their efficacy in this context.

## Materials and methods

This study included 79 rectal cancer patients over a one-year period, from 2022 to 2023. Exclusion criteria included those who had fully recovered from their chemo-radiation course and were no longer candidates for surgery, those who were eligible for local excision via the anus, the existence of neurological diseases, and those who experienced obstruction symptoms following surgery. Patients underwent rectoscopy and further imaging for staging, and those who were not eligible were excluded. Patients were randomly assigned to one of two groups: treatment group A (40 patients) or control group B (39 patients).

The study was authorized by our institution's Ethics Committee, and all patients signed an informed consent form to participate. Patient demographics, tumor characteristics, previous medical surgeries, and whether they had received chemotherapy or radiotherapy were among the information gathered. Before intervention, all patients underwent a thorough physical examination and history, completed a modified questionnaire, and any prior urinary dysfunction was documented. All of these processes were repeated immediately prior to surgery. Also, at this stage, we taught the patients using a variety of techniques, including vocal explanations, palpation, visualization, and written instructions.

On the second day after surgery, after fully removing the catheter and improving post-operative pain, group A, learned how to contract a dominant pelvic muscle while supine or sitting without contracting antagonist abdominal, gluteal, or adductor muscles. This maneuver was performed three times per day, each consisting of ten rounds in which the muscles were contracted for 3–5 s and then relaxed for 3–5 s.

In control group B, only the patient's history was obtained, and the questionnaire was completed again. All patients were followed up after discharge in an outpatient clinic, and all of the aforementioned processes were repeated 14 days after surgery. These steps were repeated at one and three months post-operation. We used the Urinary Symptom Profile (USP) Questionnaire (Appendix) and relied on the results obtained 14 days following surgery.

**Fig. 1:**
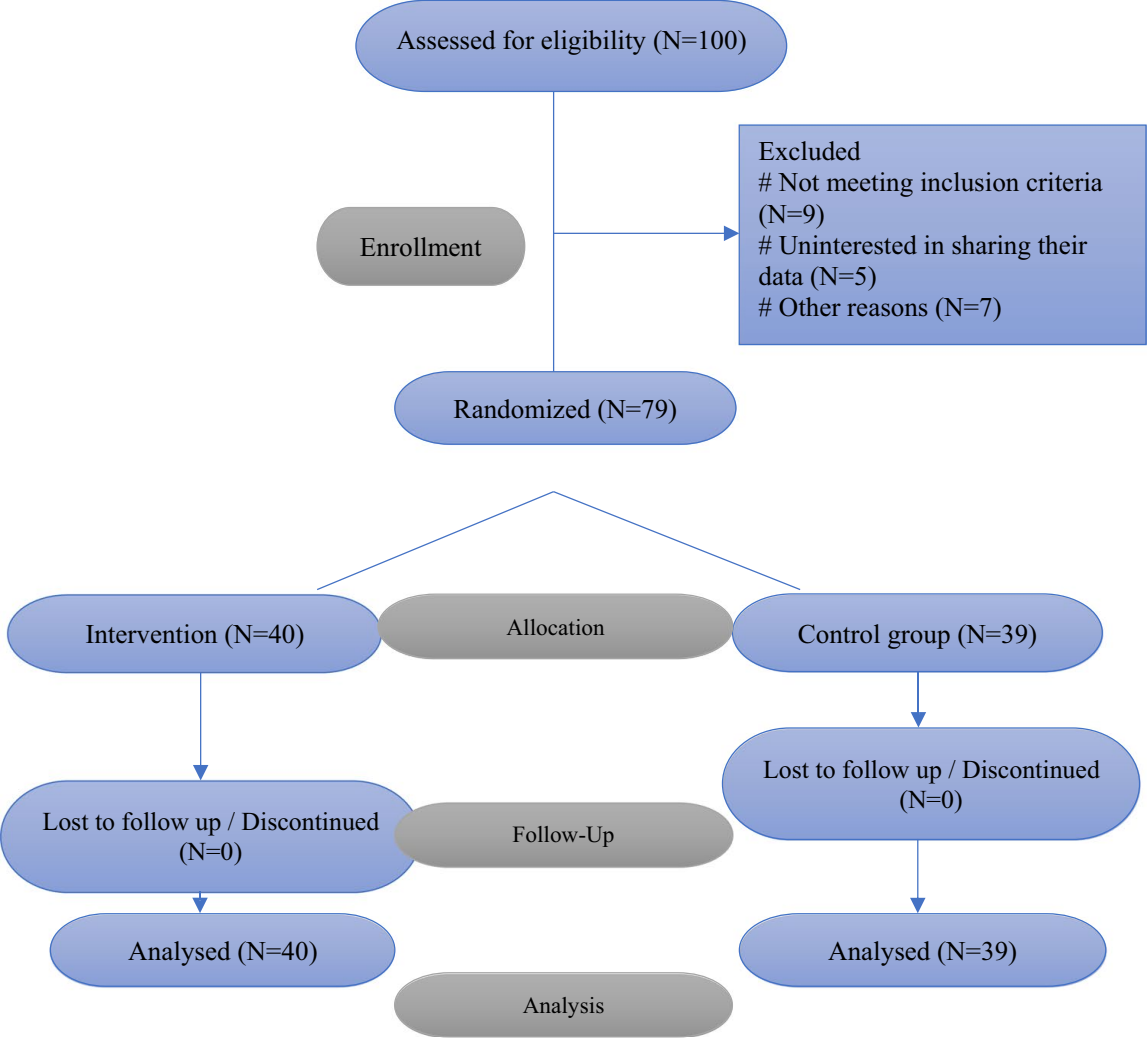
Consort flowchart

**Fig. 2:**
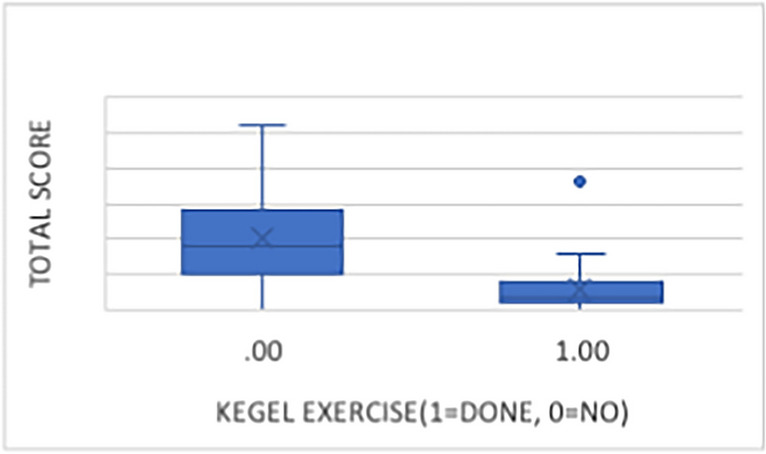
Total score relation with Kegel exercies

This 13-item assessment of lower urinary tract (LUT) symptoms is standardized and verified, addressing three domains: stress urinary incontinence (SUI) (maximum score 9), overactive bladder (OAB) (maximum score 21), and low stream (LS) (maximum score 9), with a higher score indicating worse symptoms. We used the Persian translated version of the questionnaire, which was approved our research center. Statistical analysis was conducted using IBM SPSS Statistics version 26. The t-test was employed to compare means across groups. The Pearson test was used to analyze the correlation between variables (Kegel exercises and questionnaire scores in total, as well as each part separately). Linear regression was performed to examine the association between independent variables and the dependent variable of Kegel exercises. A *p*-value of < 0.05 was considered statistically significant. Assessments were carried out as part of routine clinical care. Patients provided written informed consent for the use of anonymized questionnaire data. We have defined the main outcome of our study as the improvement in urinary function scores post-surgery. Effectiveness is considered as any statistically significant improvement in these scores (Figs. 1 and 2).

## Results

### Participant characteristics

A total of 79 rectal cancer patients were included in the study, with 40 patients allocated to treatment group A (mean age 52.9; median 52; range 20–81) and 39 patients to control group B(mean age 55; median 56; range 20–81). Baseline demographic and clinical characteristics were comparable between the two groups.

### Intervention effectiveness

Participants in treatment group A received instructions and training on Kegel pelvic floor muscle exercises, while those in control group B received standard postoperative care without specific intervention.

### Urinary symptom scores

Mean Urinary Symptom Profile (USP) questionnaire scores for stress urinary incontinence (SUI), overactive bladder (OAB), and low stream (LS) were significantly lower in group A compared to group B. In group A, mean scores were 0.32 (SD: 1.02) for SUI, 2.2 (SD: 1.8) for OAB, and 0.32 (SD: 0.85) for LS, while in group B, mean scores were 1.33 (SD: 1.96) for SUI, 7.28 (SD: 4) for OAB, and 1.1 (SD: 1.65) for LS. The total USP score was also significantly lower in group A (2.92, SD: 3.13) compared to group B (10.1, SD: 6.46). These differences were statistically significant (*p* < 0.05), with *p*-values less than 0.001 for all variables except LS.

### Correlation analysis

Linear regression analysis demonstrated a significant negative correlation between adherence to Kegel exercises and USP questionnaire scores for SUI (ß =  − 0.311, *p* = 0.005), OAB (ß =  − 0.63, *p* < 0.001), LS (ß =  − 0.288, *p* = 0.01), and total USP scores (ß =  − 0.584, *p* < 0.001). However, there was no significant association between BMI, age, gender, or type of surgery and questionnaire scores (*p* > 0.05).

### Adverse events

No significant adverse events related to the Kegel exercise intervention were reported during the study period. Participants tolerated the intervention well, with no instances of exacerbation of urinary symptoms or other complications (Tables 1 and 2).

**Table 1:** Patient characteristics

	Group A (*N* = 40)	Group B (*N* = 39)	*P*-value	95% CI for difference
Gender	65% Male, 35% Female	53.8% Male, 46.2% Female	0.31	-
Previous history of surgery	32.5% Yes	23.1% Yes	0.36	-
Mean BMI (Pre-operation)	25.21	25.02	0.78	[− 0.77,1.17]
Type of surgery (LAR/APR)	36 pt. LAR	36 pt. LAR	-	-
Tumor height mean from anal verge	7 cm	7.425	-	-
USP SUI score 14 days after surgery (mean, SD; median, IQR)	0.32(1.02); 0(0–0)	1.33(1.96); 0(0–2)	< 0.001	[− 1.702, − 0.318]
USP OAB score 14 days after surgery (mean, SD; median, IQR)	2.2(1.8); 2(1–3)	7.28(4); 7(4–10)	< 0.001	[− 6.454, − 3.706]
USP LS score 14 days after surgery (mean, SD; median, IQR)	0.32(0.85); 0(0–0)	1.1(1.65);0(0–2)	0.04	[− 1.362, − 0.198]
USP TOTAL score 14 days after surgery (mean, SD; median, IQR)	2.92(3.13); 2(1–4)	10.1(6.46); 9(14–5)	< 0.001	[− 9.427, − 4.933]

**Table 2:** Tumor characteristics (pts: patients)

	Group A (*N* = 40)	Group B (*N* = 39)
T stage	2 = 3 pts	2 = 3 pts
	3 = 25 pts	3 = 28 pts
	4 = 12 pts	4 = 8 pts
N stage	0 = 10 pts	0 = 5 pts
	1 = 20 pts	1 = 24 pts
	2 = 10 pts	2 = 10 pts
M stage	0 = 37 pts	0 = 37 pts
	1 = 3 pts	1 = 2 pts

## Discussion

Symptoms experienced by patients encompass various domains of lower urinary tract (LUT) symptoms [8]. Our study investigated the effect of early initiation of pelvic floor muscle (PFM) exercises post-surgery on reducing urinary complications in rectal cancer patients. Our data show a significant correlation between PFME and improved urinary outcomes, particularly in reducing symptoms of overactive bladder (OAB) (ß =  − 0.63, *p* < 0.001).

Comparing our findings with existing literature, our study aligns with the results of Lange et al., who emphasized the predictive value of new incontinence shortly after total mesorectal excision (TME) for persistent incontinence [9]. This underscores the importance of proactive measures, such as PFME, to mitigate such sequelae. Furthermore, our study builds upon earlier research by demonstrating the efficacy of PFME specifically in rectal cancer patients post-surgery, filling a gap in the literature.

Additionally, our study contributes to the understanding of surgical advances in rectal cancer management. With the adoption of TME procedures, the rate of urinary dysfunction has significantly decreased from 40–60% to less than 5% [10–12]. Our findings support the success of TME procedures in preserving the autonomic nervous system and reducing urinary complications. All figures were below the common cut-off threshold of the USP questionnaire different domains (SUI, OAB, LS) for indicating urinary dysfunction in both groups.

While previous studies have highlighted the influence of patient age and gender on urinary symptoms, our investigation did not find a significant association between these variables and overall test outcomes. This discrepancy may be attributed to variations in patient populations and study methodologies [13, 14].

Despite the strengths of our study, including the use of the Urinary Symptom Profile (USP) questionnaire and comprehensive assessment of LUT symptoms, certain limitations should be acknowledged. These include the relatively short follow-up period, which may limit our ability to capture long-term effects of PFME, and the reliance on self-reported outcomes. Additionally, the utilization of more precise urological assessments, such as perineal assessment of function (PAD) tests, could enhance the robustness of our evaluation.

Lastly, it is worth noting that existing literature often involves protocols with a higher number of PFMT sessions and more intensive patient monitoring by physical therapists, factors that could directly influence outcomes. Despite this, our study demonstrated significant differences between the two groups, suggesting the efficacy of our intervention even with a less intensive protocol.

## Conclusion

Initiating Kegel exercises on the first day following colorectal surgery demonstrated a significant improvement in all symptoms associated with urinary dysfunction. Our study stands as one of the pioneers in highlighting this potential benefit, signaling the need for further investigation, particularly with longer follow-ups. Determining whether patients undergoing colorectal surgery should routinely receive pelvic floor muscle training (PFMT) sessions warrants additional research to optimize postoperative care and enhance patient outcomes.

## Data Availability

Data of this research is available, per editor's request.

## Appendix

### Urinary symptom profile

#### Stress Urinary Incontinence (SUI)

1. Over the past 4 weeks, please specify the number of times a week you had leaks during physical effort: (0 = no urine leaks, 1 = less than one urine leak a week, 2 = several urine leaks a week, 3 = several urine leaks a day)
  - During strenuous physical effort
  - During moderate physical effort
  - During light physical effort

#### Overactive Bladder (OAB)

2. How many times a week have you had to rush to the toilet to urinate because you urgently needed to go? (0 = never, 1 = less than once a week, 2 = several times a week, 3 = several times a day)
3. When you have had an urgent need to urinate, for how many minutes on average have you been able to hold on? (0 = more than 15 min, 1 = from 6 to 15 min, 2 = from 1 to 5 min, 3 = less than 1 min)
4. How many times a week have you experienced a urine leak preceded by an urgent need to urinate that you were unable to control? (0 = never, 1 = less than once a week, 2 = several times a week, 3 = several times a day)
  - In the above case, what kind of leaks did you have? (0 = no leaks in this case, 1 = a few drops, 2 = light leaks, 3 = heavy leaks)

Over the last 4 weeks and under everyday conditions of social, professional or family life:

5. During the day, in general, how long elapsed between urinating? (0 = 2 h or more, 1 = between 1 and 2 h, 2 = between 30 min and 1 h, 3 = less than 30 min)
6. How many times on average have you woken up during the night by a need to urinate? (0 = never or once, 1 = twice, 2 = 3 or 4 times, 3 = more than 4 times)
7. How many times a week have you had a urine leak while asleep or have you woken up wet? (0 = never, 1 = less than once a week, 2 = several times a week, 3 = several times a day)

#### Low Stream (LS)

8. How would you describe your usual urination over these past 4 weeks? (0 = normal, 1 = needed to push with abdominal (stomach) muscles or lean forward (or required a change of position) to urinate, 2 = needed to press on the lower stomach with my hands, 3 = used a catheter)
9. In general, how would you describe your urine flow? (0 = normal, 1 = weak, 2 = drop by drop, 3 = used a catheter)
10. In general, how has your urination been? (0 = normal and quick, 1 = difficult to start then normal, 1 = easy at first but slow to finish, 2 = very slow from start to finish, 3 = used a catheter)
